# Multidimensional query processing algorithm by dimension transformation

**DOI:** 10.1038/s41598-023-31758-7

**Published:** 2023-04-11

**Authors:** Rejwana Tasnim Rimi, K. M. Azharul Hasan, Tatsuo Tsuji

**Affiliations:** 1 Department of Computer Science and Engineering, Khulna University of Engineering & Technology, Khulna, 9203 Bangladesh; 2 Faculty of Engineering, University of Fukui, Fukui-shi, Japan

**Keywords:** Computational science, Computer science, Information technology, Scientific data

## Abstract

Multidimensional query processing is an important access pattern for multidimensional scientific data. We propose an in-memory multidimensional query processing algorithm for dense data using a higher-dimensional array. We developed a new array system namely a Converted two-dimensional Array (C2A) of a multidimensional array of dimension n ($n > 2$) where the *n* dimensions are transformed into 2 dimensions. Using the C2A, we design and analyze less complex algorithms that show improve performance for data locality and cache miss rate. Therefore, improved performance for data retrieval is achieved. We demonstrate algorithms for single key and range key queries for both Traditional Multidimensional Array(TMA) and C2A. We also compare the performance of both schemes. The cost of index computation gets high when the number of dimensions increases in a TMA but the proposed C2A based algorithm shows less computation cost. The cache miss rate is also lower for in C2A based algorithm than TMA based algorithm. Theoretical and experimental results show that the performance of C2A based algorithm outperforms the TMA-based algorithms.

## Introduction

In many scientific and industrial applications, massive volumes of data have been generated, causing management and processing bottlenecks^1^. The majority of these data are multidimensional which are stored and analyzed using a multidimensional array for example SciDB^2^, SparkArray^3^, SanssouciDB^4^, and SharkDB^5^. Therefore, the efficient design of retrieval algorithms from multidimensional arrays to handle the high dimensional data is a cramming need for data scientists^6^. Traditional Multidimensional Array (TMA) facilitates quick random access to data via the addressing function, but as the number of dimensions grows, the performance of the multi-dimensional array retrieval declines. When the number of dimensions of a multidimensional array increases the cache miss rate and index computation cost automatically increase.The cache miss rate increases for higher-dimensional arrays as more cache lines need to be accessed^7,8^. Therefore, to provide fast retrieval, well organization of higher dimensional data is necessary so that the target data are colocated. In this paper, we construct and analyze a proficient retrieval strategy using dimension transformation. We convert an *n* dimensional TMA into a two-dimensional array namely a Converted two-dimensional Array (C2A). The C2A represents an *n* dimensional ($n>2$) array by a 2-dimensional array where each odd dimension of the TMA contribute for *row* and each even dimensions contribute for *column* dimension. With the dimension transformation, the target data are colocated which helps to design less complicated algorithms for efficient retrieval. The superiority of transformation is well studied in the loop transformation technique for compiler optimization^9^ and higher dimensional matrix operations^10^. The array cells are rearranged by changing the loop nests to make them closer to the cache memory in the loop transformation. This transformation is useful for array operations to increase the cache hit rate. In our approach, when we convert the *n* dimensional TMA to a 2 dimensional C2A, the *n* loops are transformed into two loops namely outer and inner loops. As a result, enhanced data locality is possible since the inner loops randomly access the cache, which increases cache misses. We apply the C2A scheme to design new retrieval algorithms for array-based multidimensional data. Therefore, improved retrieval performance is found with the proposed C2A-based algorithms. Our theoretical and experimental analysis shows that with the growth of a number of dimensions, the C2A-based algorithms outperform the TMA-based algorithms. The multidimensional query is well defined for datacube computation as MOLAP operations^11^. Therefore it can easily be applied to datacube computation^12,13^. The scheme can also be applied to multidimensional databases, Top-*k* queries^1,14^, and multiway data analysis^15^. The rest of the paper is organized is as follows: firstly explain the idea of dimension transformation, then retrieval algorithm for multidimensional query processing, reveals the theoretical analysis of the algorithms, and then show the experimental results, some comparisons with other works are presented in related works and finally, outlines the conclusion.

## Dimension transformation

Let $A[S_1][S_2]\ldots [S_n]$ be a TMA(n) where $< x_1, x_2, \ldots , x_n>$ be the index of an element of A and $S_1, S_2, \ldots , S_n$ are the size of each dimension $d_1, d_2,\ldots , d_n$ and $x_i = 0, 1, 2, 3, \ldots , S_{i-1}$ where $1 \le i \le n$ . Any element of TMA(n) $< x_1, x_2,\ldots , x_n>$ can be accessed by the addressing function $f(x_1, x_2, \ldots , x_n)=x_1 S_2 S_3 \ldots S_n+ x_2 S_3S_4 \ldots S_n+\cdots +x_{n-1} S_n + x_n$. We develop a two-dimensional array C2A $A'[X'][Y']$ of size $S_1', S_2'$ and subscripts $<X', Y'>$ where $X'(0\le X' < S_1')$ and $Y(0 \le Y' \le S_2')$. The $X'$ and $Y'$ are converted as follows :

$$\begin{aligned} \begin{aligned} X'= \left\{ \begin{array}{l} x_1 S_3 S_5\ldots l_{n-3} S_{n-1 }+x_3 S_5\ldots S_{n-3 }S_{n-1}+\cdots +x_{n-3 } S_{n-1 }+x_{n-1}, \; \; when\; n \; is \; even.\\ x_1 S_3 S_5\ldots l_{n-2 } S_n + x_3 S_5 S_7\ldots S_{n-2}S_n+\cdots +x_{n-2} S_n+x_n,\;\; \; when\; n\; is \; odd. \end{array} \right. \\Y'= \left\{ \begin{array}{l} x_2 S_4 S_6\ldots l_{n-3}S_{n-1}+x_4 S_6\ldots l_{n-3}S_{n-1}+\cdots +x_{n-3}S_{n-1}+x_{n-1}, \; \; when\; n \; is \; even.\\ x_2 S_4 S_6\ldots S_{n-2}S_n+ x_4 S_6 S_8\ldots S_{n-2} S_n+\cdots +x_{n-2}S_n+x_n,\;\; \; when\; n\; is \; odd. \end{array} \right. \end{aligned} \end{aligned}$$

From the above equation,the four-dimensional array is converted as $X'= x_1 S_3+ x_3$ and $Y'= x_2 S_4+ x_4$. Similarly, the six dimensional array is converted to C2A as $X'= x_1 S_3 S_5+ x_3 S_5+x_5$ and $Y'= x_2 S_4 S_6+ x_4 S_6+x_6$. Therefore, any element of C2A $<X', Y'>$ can be found by the addressing function:

$$\begin{aligned} \begin{aligned} f(X',Y')=X'S_2'+ Y' \ or \ f(X',Y')=Y'S_1'+ X' \end{aligned} \end{aligned}$$

where

$$\begin{aligned} \begin{aligned} S_1'= \left\{ \begin{array}{l} S_1 \times S_3 \times S_5 \times \cdots \times S_{n-1 }, \;\; \; when\; n \; is \; even.\\ S_1 \times S_3 \times S_5 \times \cdots \times S_{n } \;\; \; when\; n\; is \; odd. \end{array} \right. \\S_2'= \left\{ \begin{array}{l} S_2 \times S_4 \times S_6 \times \cdots \times S_{n}, \;\; \; when\; n \; is \; even.\\ S_2 \times S_4 \times S_6 \times \cdots \times S_{n-1}, \;\; \; when\; n\; is \; odd. \end{array} \right. \end{aligned} \end{aligned}$$

**Figure 1:**
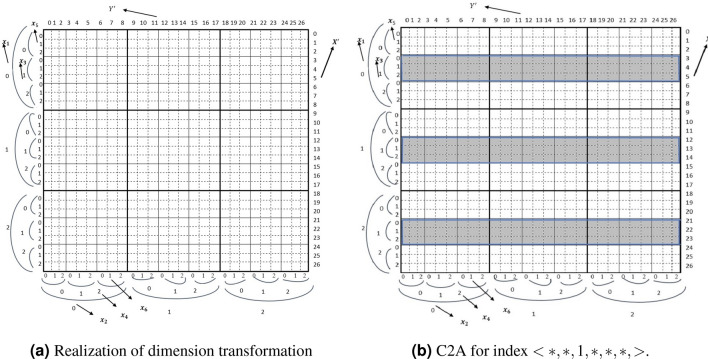
Single key realization of C2A for $X'$.

Figure 1a shows a converted TMA of size^3^ to C2A of size^27^. We describe the C2A with odd dimensions as *row* and even dimensions as *column*. This *row* and *column* can be selected from any combinations from the *n* dimensions. The arbitrary combination does not have much effect for retrieval of array data^15^.

## Multidimensional query processing algorithm

We design multidimensional query processing algorithms for single key and range key query. Answering a query *q* consists of selecting the set of points from *R* that satisfy the query predicate of the form $x_i=v$ for single key query and for $x_i=v_1\sim v_2$ or $x_i>v_1$ or $x_i<v_1$ for range key query where *R* is the dataset of dimension *n*. Each point of *R* is specified by *n* co-ordinates each of which is a member of a specific domain $d_i$ ($1 \le i \le n)$. By the dimension transformation technique (Sect. 2) the *n* dimensional point *x* is converted to 2 dimensional points $x'$ and the query is performed in the converted 2 dimensional space. Throughout the paper, we define a single key query of $x_i$ by form $<*,*,\ldots v,\ldots *,*> (0 \le v \le l_i -1)$ and $<*,*,\ldots v_1\sim v_2,\ldots *,*>$ for range key query, where $v_1$ and $v_2$ $(v_1>v_2)$ is the value of the index $x_i$ and $d_i$ is termed as known dimension and $rq=\mid v_1 - v_2 \mid +1$.

### Single key query

Let $A [S_1] [S_2 ] [S_3] [S_4]$ be a TMA (4) of size $[S_1, S_2, S_3, S_4]$. The location of the tuple $A[x_1][x_2][x_3][x_4]$ can be identified by index computation function $f(x_1, x_2, x_3,x_4)$. The retrieval from TMA is straightforward. Algorithm 1 shows the pseudo code of single key query for the tuple $<v,*,*,*\ldots>$ for TMA(n) where $x_1=v$. We need $(n-1)$ loops to carry out the search. In the next subsections we derive the algorithm to retrieve from C2A. Figure 1 shows the candidate array cells for a single key query.
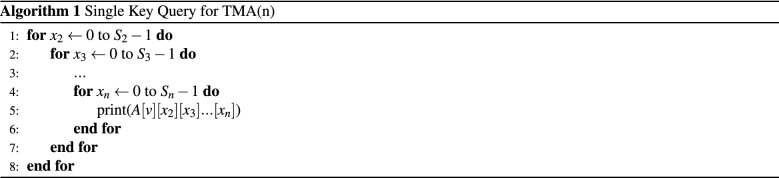


#### C2A algorithm development

Since C2A is a two-dimensional structure, it will take two loops only to retrieve any element. Following parameters are important to retrieve an element from C2A. The algorithm is designed to calculate these parameters.

- value Start Index (SI) for $x'$ ( or $y'$)
- total number of Target Rows (TR) (or Target Columns) for retrieval operation.
- striding values to continue the loops.

We need three types of indices for TMA namely *inner index*, *outer index* and *intermediate index* for C2A algorithm development. Figure 1 shows the three types indices for $< x_1,x_3,x_5>$ where $x_1$ is the *outer index*, $x_3$ is *intermediate index* and $x_5$ is the *inner index*. The *intermediate index* for a TMA(n) ($n\le 4$) is void. We will present algorithms for C2A for four-dimensional (4D) array and then extend it to *n* dimensional arrays.

**Figure 2:**
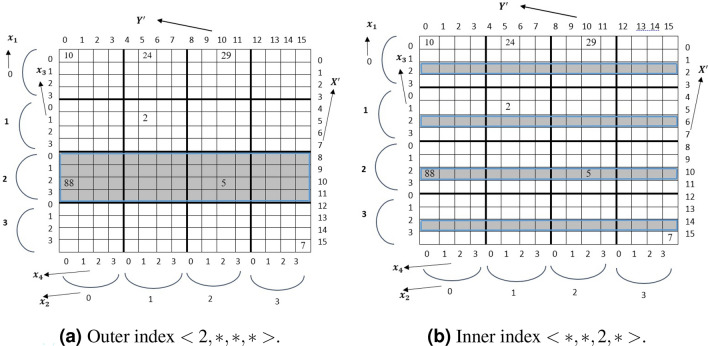
Single key realization of C2A for $X'$.

**4D:** For a query of the form $x_i=v$ where *i* is odd and $(1 \le i \le 4 )$ . For example, to retrieve the tuple $<2,*,*,*>$ which is the *outer index* as shown in Fig. 2a. The candidate rows for the query are $X'=<8, 9, 10,11>$. Hence $SI=8$ and $TR=4$. This candidate rows can be found in unit striding (i.e. striding value is 1). If $x_i$ of dimension *i* is known, then the $SI = x_i\times S_3=8$ and $TR= \prod _{p=1,3} S_p (p\ne i)$. In case of tuple $<*,*, 2,*>$ (see Fig. 2b) where $x_3$ is known. The *SI* for C2A will be 2 (i.e $x_3$) because $x_3$ is an *inner index*. The stride value is $S_3$. Therefore, candidate row indexes will be $X'=<2,6, 10, 14>$.

Again for a query of the form $x_i=v$ where *i* is even and $(1 \le i \le 4 )$, the algorithm returns column index. For the tuple $<*,2,*,*>$. The *SI* for C2A is be $x_2 \times S_4 =2 \times 4 = 8$ and the stride value is 1. The candidate column indices for the query are $Y'=<8,9,10,11>$. And for the tuple $<*,*,*,2>$ is the fourth index, the *inner index*, of TMA. The stride value for the loop is $S_4$. Therefore, the candidate indices are $Y'=<2, 6, 10, 14>$. Algorithm 2 summarizes the query processing for the converted row index $X'$.
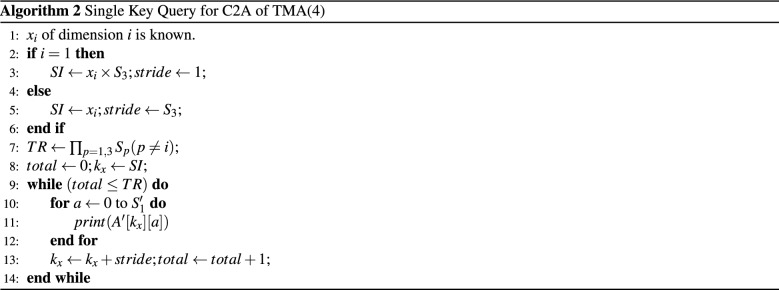


**6D:** Let $A[S_1][S_2][S_3][S_4][S_5][S_6]$ be a TMA(6) of size $[S_1 S_2 S_3 S_4 S_5 S_6]$. After transforming *A* to $A'$, the subscript of a tuple $<x_1,x_3,x_5>$ contributes for $X'$ and $<x_2,x_4,x_6>$ contributes for $Y'$. For example, for the query $<*,*, 1,*,*,*>$ $x_3$ is known. Because the known index is intermediate index, the SI for C2A will be $x_3 \times S_5$ and to retrieve the this index it needs 2 types of stride values namely *unit stride* and *long stride*. There are some consecutive target rows that are in unit stride and there also a period between the consecutive target rows which is called *long strides* (Fig. 1). For example, the target rows are $X'=<3-5, 12-14, 21-23>$ for the query of the tuples $<*,*, 1,*,*,*>$ as shown in Fig. 1. The consecutive ($x_3 \times S_5$) indices can be found by unit striding. The *long stride* between two consecutive *unit stride* is determined by $p_i=S_5 \times (S_3 -1)$. The *long stride* is determined by the computation procedure of $X'$ as described in Sect. 2.

The summary of the query processing is shown in Algorithm 3 for $X'$.

**nD:** Let $A[S_1][S_2]\ldots [S_n]$ be a TMA(n) of size $[S_1 S_2 \ldots S_n]$ and $x_i$ of dimension $i (1 \le i \le n )$ is known. The values for SI of C2A for the known dimension *i* can be determined as $SI=x_i$ for *inner index*, $SI=x_i \times S_{3} \times S_{5} \times \cdots \times S_{n-1}$ for *outer index*, $SI=x_i \times S_{5} \times S_{7} \times \cdots \times S_i$ for *intermediate index*. The stride values for *outer* and *inner index* can be determined as $stride=S_{n-1}$ for *inner index*, $stride =1$ for *outer index*, The stride values depend on the known dimension. There are $(n/2-2)$ intermediate indices possible (See $X'$ computation).
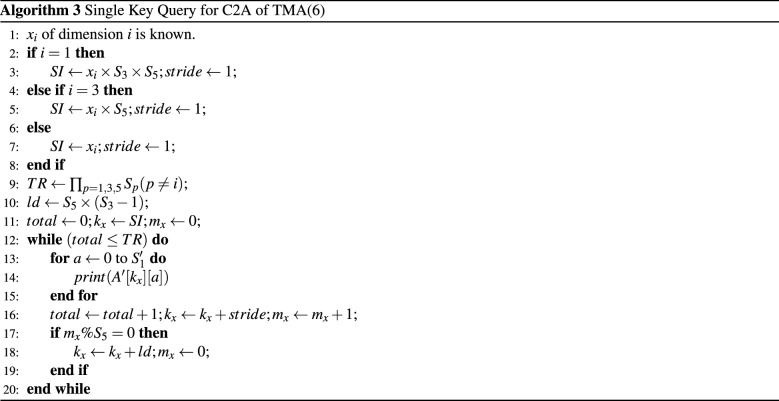


### Range key query

Let the range value of a range key query is $nrq=v_2-v_1, (v_2>v_1)$. Therefore, the total target rows will be $TRQ=nrq \times \prod _{p=1,3,5} S_p (p\ne i)$ for a C2A. Suppose we want to consider a query of $<0-1,*,*,*>$ for TMA(4). The target rows for the query are $X'=<0-7>$. The $SI =v_1 \times S_3$,$TRQ=rq\times S_3$ and $rq=(v_2-v_1)+1$ ($SI=0$ and $TRQ=8$). And unit striding is possible. If $d_i$ is the known for 4D case, the $SI =v_1\times S_3=4$ and $TRQ=nrq\times \prod _{p=1,3} S_p (p\ne i)$. For example, for the query of the tuple $<*,*, 0-1,*>$, the *SI* for C2A will be 1 (i.e $x_3$) because $x_3$ is an *inner index*. The stride value is $S_3-nrq$. Therefore, the row indices will be $X'=<0-1,4-5, 8-9, 12-13>$.

**Figure 3:**
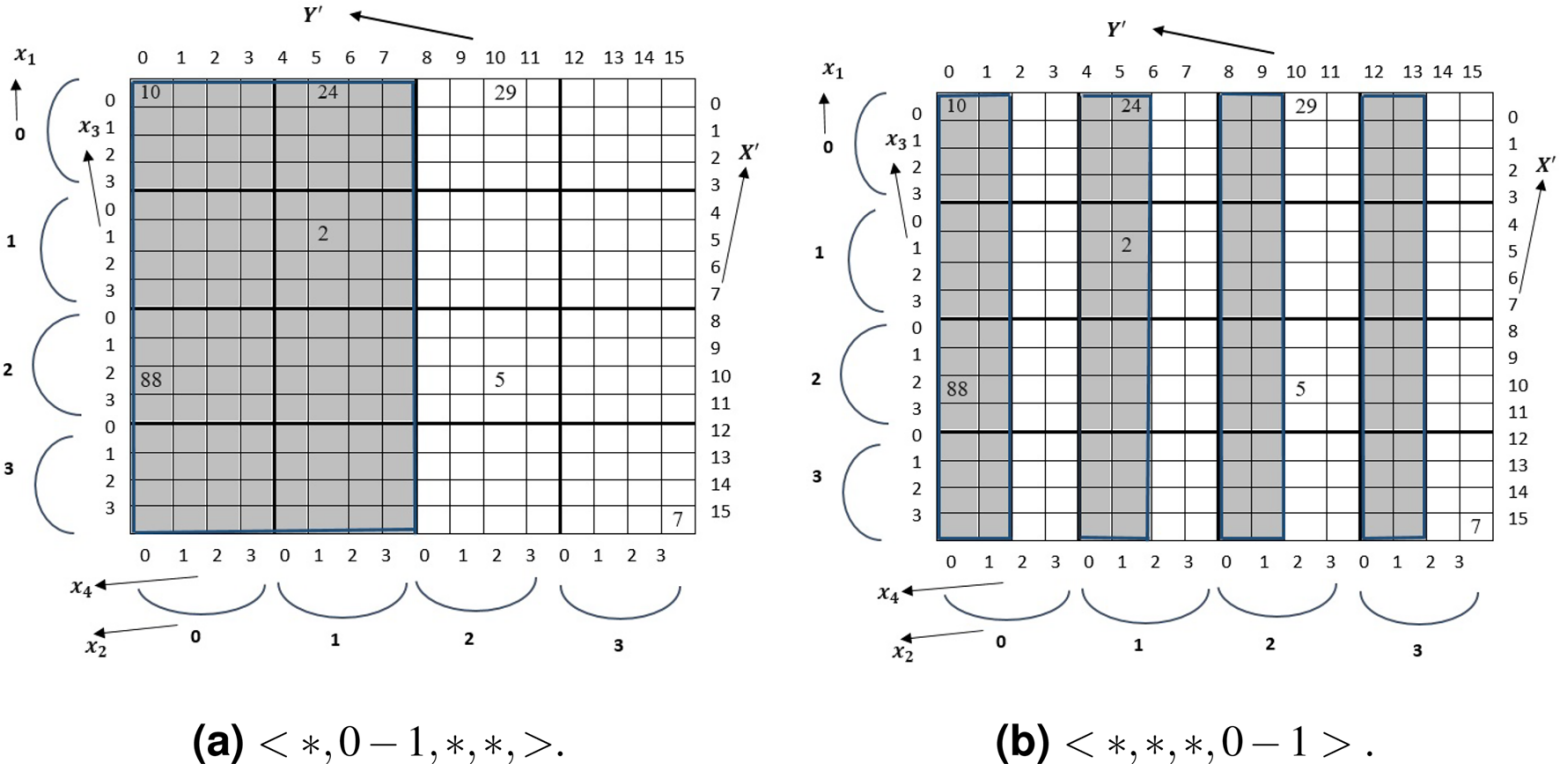
Range key query realization by C2A for $Y'$.

Again, for the query of the tuple $<*,0-1,*,*>$, the SI and will be calculated as $v_1 \times S_4 =0 \times 4 = 0$ and stride value is 1. The candidate column indices are $Y'=<0-7>$(see Fig. 3a). For the tuple $<*,*,*, 0-1>$ the stride value is $(S_4-nrq)$. Therefore, the target column indices are $Y'=<0-1,4-5, 8-9, 12-13>$ (Fig. 3b).

**Figure 4:**
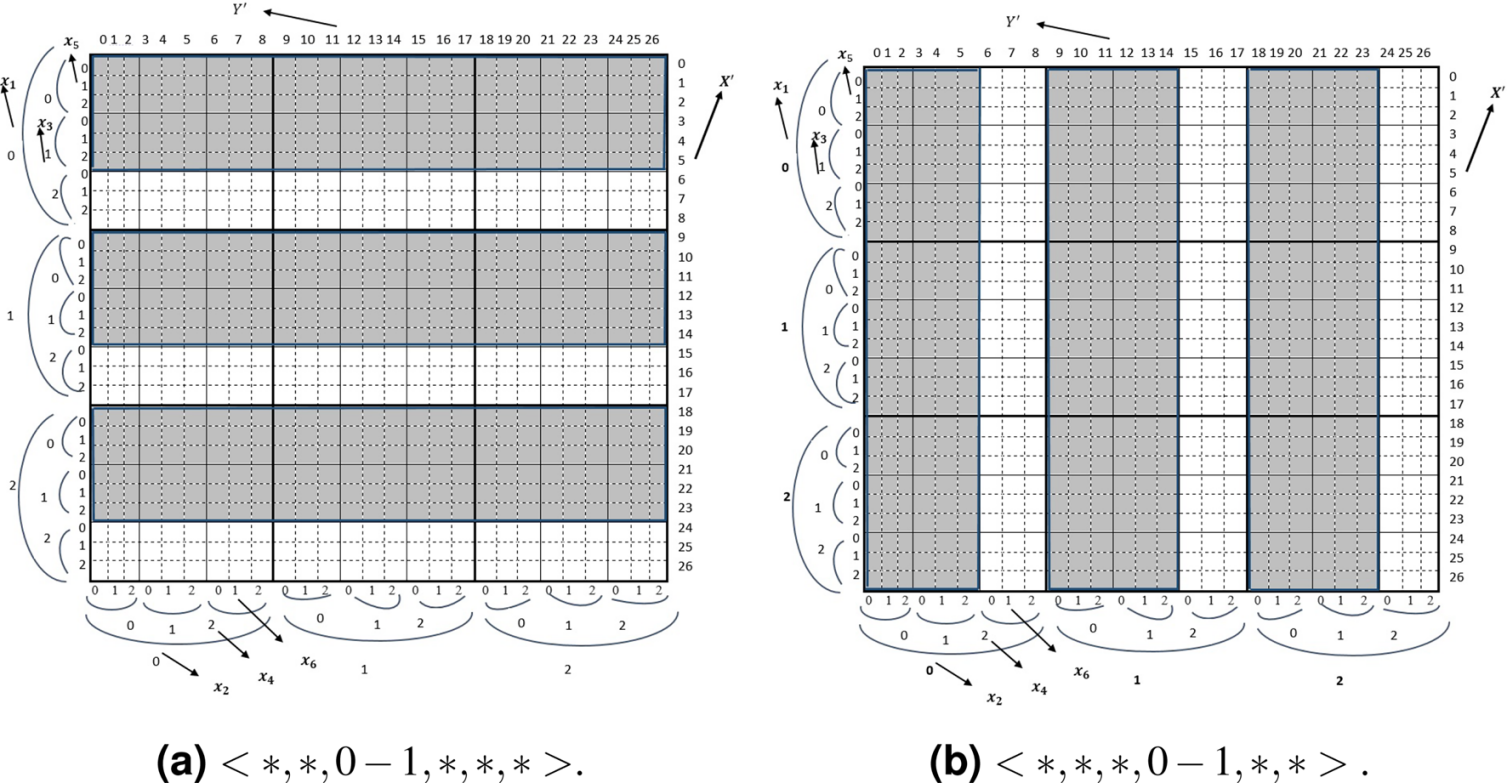
Range key query realization by C2A.

The candidate range of rows for the query $<*, *, 0-1,*,*,*>$ is shown in Fig. 4. For the query $<*,*, 0-1,*,*,*>$ $x_3$ is known and the *SI* for C2A will be calculated as $v_1 \times S_5$. The query has both *unit stride* and *long stride* as the known index is an intermediate index. In Fig. 4a for the query $<*,*, 0-1,*,*,*>$ where the target rows are $<0-5, 9-14,18-23>$. There are 3 consecutive target rows ($TRQ= S_5\times nrq$) that can be found by unit striding. The *long stride* between two consecutive rows is determined by $p_i=S_5 \times (S_3-nrq)$.

For a nD TMA $A[S_1][S_2]\ldots [S_n]$ of size $[S_1 S_2 \ldots S_n]$, the values for *SI* and the parameters are calculted as *inner index*, $SI=v_1$, for *outer index* and *intermediate index* $SI=v_1 \times S_{n-1} \times l_{n-3} \times \cdots \times l_{i+2}$. The stride is $S_{n-1}-nrq$ for *inner index* and 1 for *outer index* again for *intermediate index*, long stride is $ld=S_{n-1} \times \cdots \times S_7 \times S_5 \times (S_3 - nrq)$ and unitt stride. The algorithm for C2A of TMA(n) is summarized in Algorithm 4 for row indices $X'$.
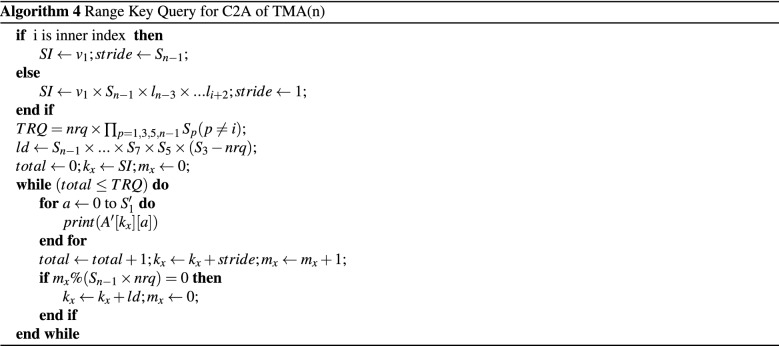


## Theoretical analysis

Three aspects are considered for theoritical analysis namely *cost for index computation*, *cost for cache line access* and *computational complexity*. We assume the length of dimension is equal for each TMA dimensions ($S_i =S$ for $1\le i \le n$). The total number of addition and multiplication operations contributes to index computation. Let $\alpha$ be the cost of multiplication, $\beta$ be the cost of addition operation and $\eta$ be the improvement of C2A based algorithm over TMA based algorithm. We developed the theoretical analysis for a single key query in this section. The theoretical analysis for a range key query is a straightforward extension of a single key query because when $nrq=1$ it becomes a single key query.

### Cost for index computation

For a single key query, the number of elements to be accessed is $S^{n-1}$ both for TMA and C2A. The number of elements to be accessed is $nrq\times S^{n-1}$ for the range key query. These elements are accessed only once.

**4D:** The index computation function of TMA and C2A are *f* and $f'$ $f(x_1, x_2, x_3,x_4)=S\times S\times S \times x_1+S\times S \times x_2+S \times x_3 + x_4$ and $f'( X', Y')=X'\times S_1' +Y'$ where $X'= x_1 \times S_3+ x_3$, $Y'= x_2 \times S_4+ x_4$ and $S_1'=S_1\times S_3$. Therefore, *f* require 6 multiplication ( $6\alpha$) and 3 addition ($3\beta$) operations. Hence the cost for *f* is $(6\alpha +3\beta )S^3$. On the other hand, $f'$ require 1 multiplication and 1 addition opertations resulting the cost for $f'$ is $(\alpha +\beta )S^3$. The transformation cost for $X'$ is $\alpha +\beta$ and $Y'$ is $\alpha +\beta$. And $S_1'$ is $\alpha$. All the transformations do not require any element to access. And these transformations are done only once. Therefore, total cost for C2A is $(\alpha +\beta )S^3+ 3\alpha +2\beta$. Hence, $\eta = (1-\frac{(\alpha +\beta ) S^3+3\alpha +2\beta }{(6\alpha +3\beta )S^3}) \times 100\%$. We can simplify the equation by considering $(\alpha>>\beta )$ as Multiplication latency (IMUL) is 3 to 15 times longer than addition latency (ADD). We can also ignore the $\beta$ with respect to $\alpha$, then $\eta = (\frac{5}{6}-\frac{3}{6S^3})\times 100\%$.

**6D:** There are 15 multiplication operations and 5 addition operations required for *f*, hence, the cost for *f* is $(15\alpha +5\beta )S^5$. And $f'$ requires 1 addition and 1 multiplication and cost is $(\alpha +\beta )S^5$ . The transformation cost for $X'$ is $3\alpha +2\beta$ and $Y'$ is $3\alpha +2\beta$. And transformation cost for $S_1'$ is $2\alpha$. Therefore, total cost for C2A is $(\alpha +\beta ) S^5+ 8\alpha +4\beta$. Therefore, $\eta =( 1-\frac{(\alpha +\beta ) S^5+8\alpha +4\beta }{(15\alpha +5\beta )S^5 }) \times 100\%$, and $\eta = (\frac{14}{15}-\frac{8}{15S^5})\times 100\%$ for $\alpha>>\beta$.

**nD:** For nD TMA, *f* need $(n-1)$ addition and $\frac{(n(n-1))}{2}$ multiplication operations. Therefore, costs are $(n-1)\beta$ and $\frac{(n(n-1))}{2} \alpha$ respectively. So total cost for TMA is $\frac{((n(n-1))}{2} \alpha + (n-1) \beta )S^{n-1}$. The transformation cost for is $\frac{n(n-2)}{4}\alpha$, $((n/2)-1)\beta$. Therefore,

$$\begin{aligned} \eta = (1-\frac{(\alpha +\beta ) S^{(n-1)}+\frac{n(n-2)}{4} \alpha + 2 \times (\frac{n}{2}-1)\beta }{(\frac{n(n-1)}{2} \alpha +(n-1)\beta )S^{n-1}}) \times 100\% \end{aligned}$$

For $\alpha>>\beta$, $\eta = (1-\frac{2}{n(n-1)}-\frac{(n-2)}{2(n-1)S^{n-1}})\times 100\%$. Therefore, we conclude that, for large values of *n* and *S*, the $\eta$ will increase. Hence, the proposed retrieval algorithm will get the facility for large arrays for index computation.

### Cost for cache line access

The cache line is the unit of data transfer between the main memory and the cache. A whole line is read or written during data transfer by the system. The number of cache lines accessed can be determined by the algorithm namely *LoopCost*(*S*) proposed by Carr et al.^7,8^. The *LoopCost*(*S*) finds the number of cache lines accessed by a loop by computing the costs of various loop orders. We use the *LoopCost*(*S*) to analyze the cache line access of our algorithm. The value of *LoopCost*(*S*) indicates the cache miss rate for a loop. Hence smaller the value of *LoopCost*(*S*) indicates a smaller cache miss and higher cache hit. Let the cache line size be *r* (generally this size is 64 bytes). For 4D, we assume the loop order $<S_1,S_2,S_3,S_4>$ to maintain the sequential access of the memory. The cache line accessed by TMA is determined by $S^3 \lceil S/r\rceil$. Since there are three inner loops of length *S* in Algorithm 1. On the otherhand, the cache line accessed by C2A is determined by $S^2 \lceil (S^2/r)\rceil$. Since there is only one inner loop of length $S^2$ in Algorithm 2. Therefore,

$$\begin{aligned} \eta =1- \frac{C2A}{TMA} = 1- \frac{S^2 \lceil \frac{S ^2}{r})\rceil }{S^3 \lceil \frac{S}{r} \rceil } = 1- \frac{\lceil (\frac{S^2}{r})\rceil }{S\lceil \frac{S}{r} \rceil }. \end{aligned}$$

If $S\mod r=0$ then $\eta =0$, otherwise $\eta >0$. For nD, we assume $<S_1,S_2,\ldots ,S_n>$ loop order to mainatain the sequential access. The number of cache line accessed by C2A is $S^{\lceil \frac{n}{2}\rceil } \lceil { \frac{ S^{\lceil \frac{ n}{2}\rceil }}{r}}\rceil$ and the number of cache line accessed by TMA is $S^{n-1} \lceil \frac{S}{r}\rceil .$ Therefore,

$$\begin{aligned} \eta =1-\frac{ S^{\lceil \frac{n}{2}\rceil }\lceil S^{\frac{\lceil \frac{n}{2}\rceil }{ r} }\rceil }{S^{n-1} \lceil \frac{S}{r}\rceil } \end{aligned}$$

Finally, we conclude that, if *S* is divisible by *r* then $\eta =0$, i.e. the number of cache lines accessed for both schemes are the same. When *S* is not divisible by *r*,  then $\eta >0$.

**Figure 5:**
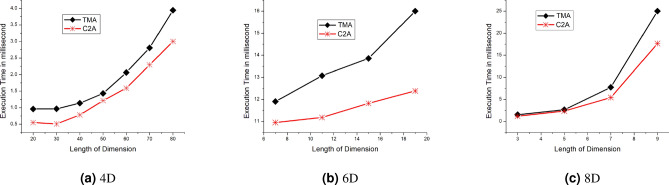
Performance of TMA and C2A for single key query for varying length of dimension for 4D, 6D and 8D.

In the case of sequential access of sparse data in memory, TMA requires more time because data are stored in a more scattered way than C2A . We know, the linear equation for TMA and C2A is, *f* and $f'$

$$\begin{aligned} f(x_1, x_2, x_3,x_4)=s_1\times s_2\times s_3 \times x_4+s_1\times s_2 \times x_3+s_1 \times x_2 + x_1 \end{aligned}$$

$$\begin{aligned} f'( X', Y')=X'\times S_2' +Y' \end{aligned}$$

where $X'= x_1 \times s_3+ x_3$, $Y'= x_2 \times s_4+ x_4$ and $S_2'=s_1\times s_3$. Let,$s_1= s_2= s_3 = s_4 =2$. For TMA, $x_1=1$

Then, $f=8 \times x_4+4 \times x_3+2 \times x_2 + 1$ ,so, $f= \{1,3,5,7,9,11,13,15\}$ and stride is $s=2$.

When $x_2=1$ Then, $f=8 \times x_4+4 \times x_3+2 + x_1$ ,so, $f= \{2-3,6-7,10,11-14-15\}$ and stride is $s\times (s-1)=3$.

When $x_3=1$ Then, $f=8 \times x_4+4 +2\times x_2 + x_1$ ,so, $f= \{4-7,12-15\}$ and stride is $s\times s\times (s-1)=5$.

When $x_4=1$, Then, $f=8+4\times x_3 +2\times x_2 + x_1$ ,so, $f= \{8-15\}$. For C2A, $f'=4\times x_2'+x_1'$

When, $x_1=1, x_1'=x_1 \times s_3+x_3, x_1'=\{2,3\}$ (Because the value of $x_3$ is 0 and 1)

Therefore, $f'=\{2-3,6-7,10-11,14-15\}$ and stride is $s\times s-1=3$.

When, $x_3=1, so, x_1'=\{1,2\}$ $f'=\{1-2,5-6,9-10,13-14\}$ and stride is $s\times s-1=3$.

When, $$x_2=1, x_2'=x_2 \times s_4+x_4, x_2'=\{2,3\}$$ $$f'=\{8-15\}$$

When, $$x_4=1, so, x_2'=\{1,2\}$$ then, $$f'=\{4-7,8-11\}$$

So, for 4D stride of TMA will be $s\times s\times (s-1)$ and for n-D it will be $s^{n-2}\times (s-1)$. And for C2A the hightest striding value for n-D will be $s^{n/2-1}\times (s-1)$ which is less than TMA. The compiler maintains the row-wise data layout in the system. That’s why row-wise retrieval has quite improved performance than column-wise retrieval.

### Computational complexity

For 4D, the computational complexity of single key query for C2A based algorithm (algorithm 2) is $O(S \times S^2)=O(S^3)$ since the *TR* iterates for *S* times because the striding values are different. For 6D, the computational complexity of C2A based algorithm (algorithm 3)is $O(S^2 \times S^3)=O(S^5)$. Therefore, the computational complexity of C2A based algorithm for nD is $O(S^{n-1})$. For range key query, $nrq \times s_i$ target rows are executed from the known dimension *i*. Hence the complexity for for range key query for C2A based algorithm is determined by $O(nrq \times S^{n-1})$.

## Experimental results

In this section, we compare the retrieval time of TMA-based algorithms versus the retrieval time of C2A based algorithms for single key query and range key query. The original data was in TMA and transformed to a C2A. We ignore the conversion cost from TMA to C2A. If the compiler provides the converted array C2A, the conversion cost can be ignored. Using the programming language C++, we take the execution time in milliseconds. We assume the array is dense.

**Figure 6:**
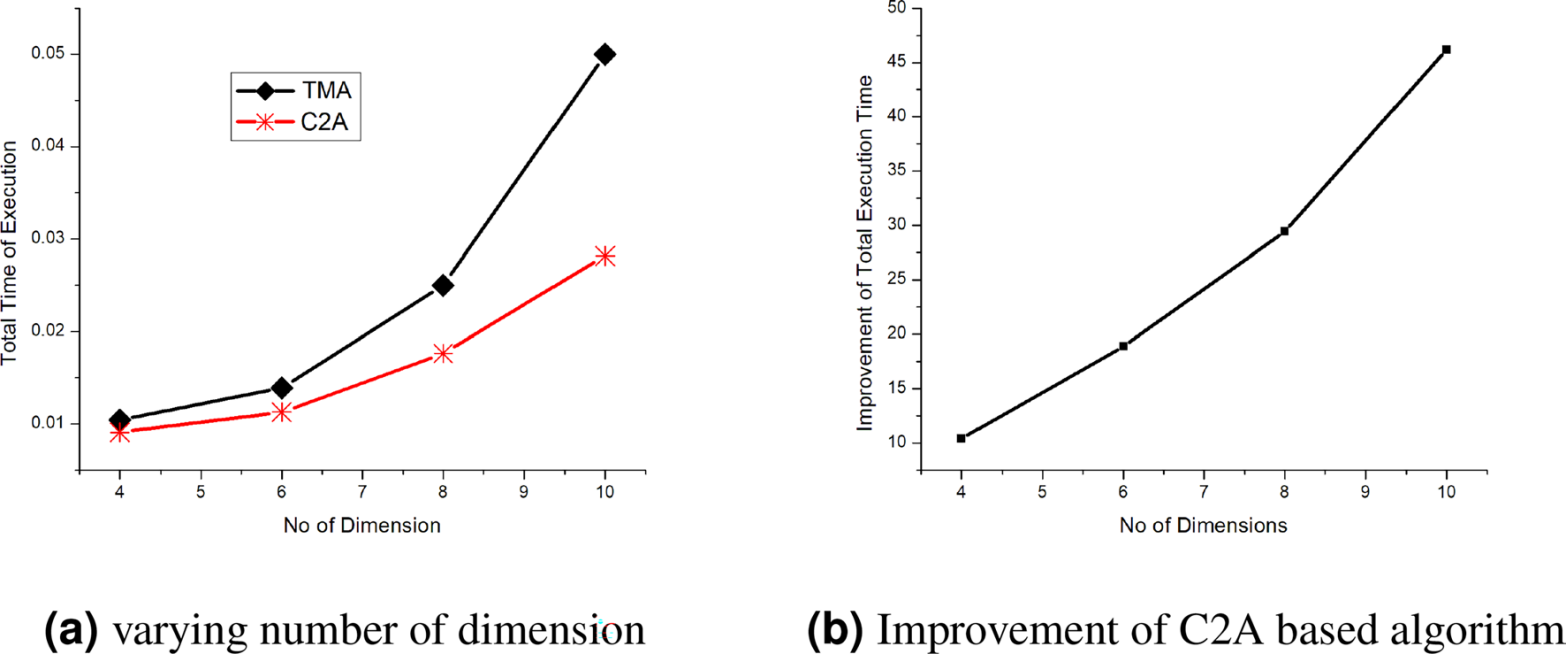
Performance analysis of 4–10 dimensions for Single key query (TMA and C2A).

**Figure 7:**
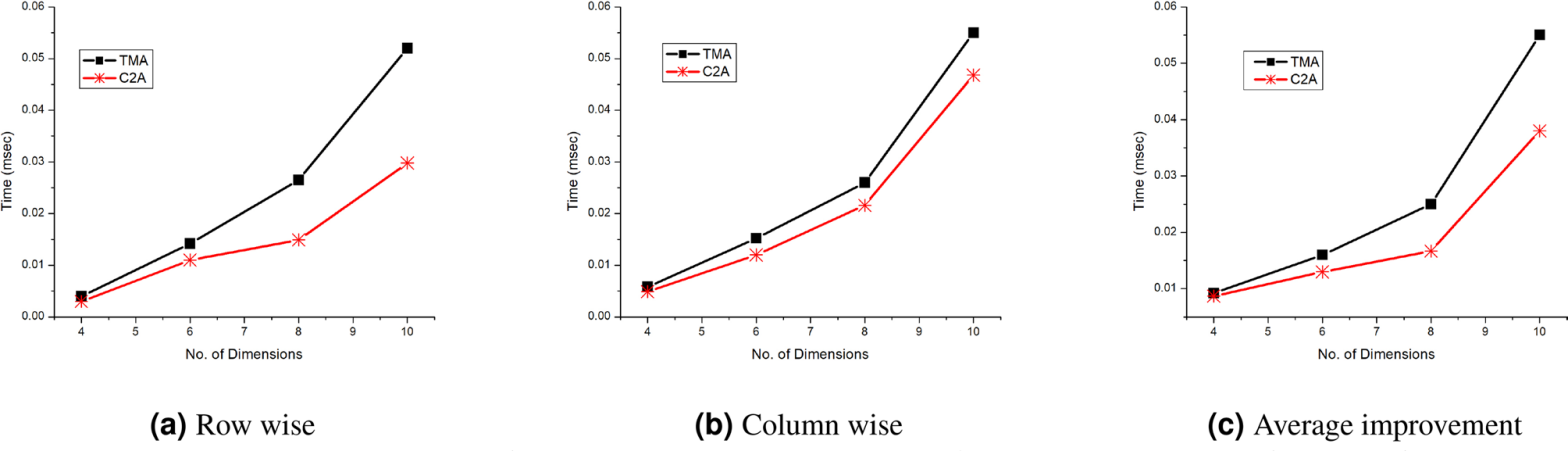
Performance of row and column wise retrieval of Single key query for TMA and C2A with varying n.

Figure 5 shows the performance comparison for TMA versus C2A for a single key query for varying lengths of dimension for 4D, 6D, and 8D. All the possible single key queries are performed and the average of the results are shown in Fig. 5. The execution time of C2A based algorithms has a clear improvement over TMA-based algorithms for all the cases in Fig. 5a,b,c. This is because when the size of dimension *S* increases, the improvement $\eta$ also increases. For large values of *S* and *n*, better performance for C2A based algorithms is found as discussed in Sect. 4. The C2A based algorithms get the advantages of less computational cost for index computation than TMA-based algorithms. Since TMA-based algorithms have many loops where C2A has only two loops. This number of loops gets increased when the number of dimension increase in TMA whereas the number of loops in C2A is fixed irrespective of the value of *n*. for example, the C2A based algorithms have 2 loops whereas the TMA based algorithms have $n-1$ loops for single key query and *n* loops for range key query. The cache miss is reduced because the C2A based algorithms have only an outer loop. But the cache miss has increased in TMA-based algorithms as it has $n-2$ (or $n-1$ range key query ) outer loops. this is because of the influence of the outer loop for random access of memory where as the influence of the inner loop to access the memory sequentially. Therefore, the cache miss rate for C2A based algorithms is lower than the TMA-based algorithms. Reducing the cache miss is desirable for the programmers and researchers as it improves the retrieval of data.

**Figure 8:**
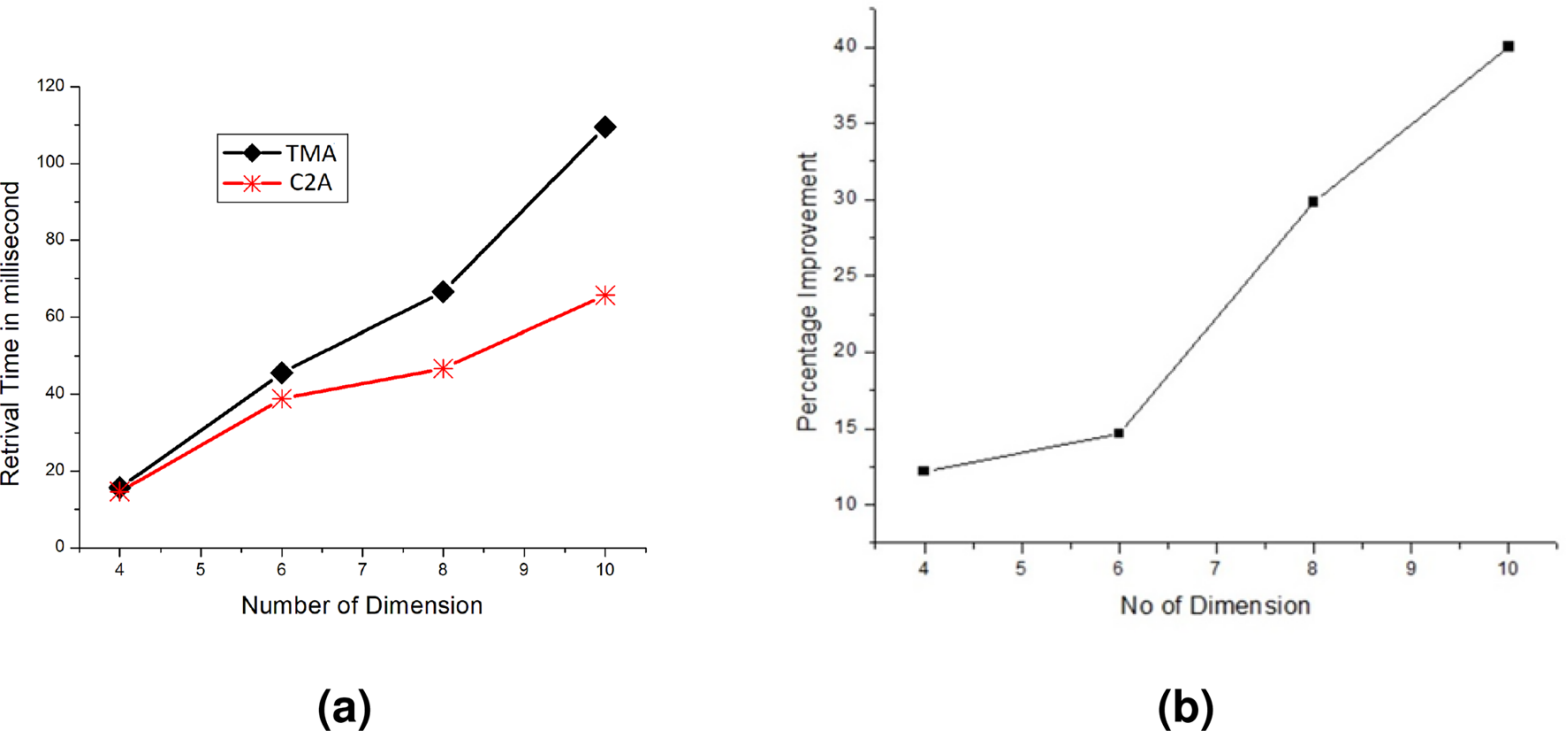
Performance analysis of 4–10 dimensions for range key query (TMA and C2A).

**Table 1:** Time comparison of single key and range key query for C2A based algorithms.

Dimensions	Single key query			Range key query		
	TMA	C2A	Improvement (%)	TMA	C2A	Improvement (%)
4D	4.014706	3.597059	10.40293	15.72917	14.69228	6.592116
6D	13.92105	11.28947	18.90359	45.57778	38.9	14.65139
8D	25.01389	17.65278	29.42809	66.625	46.75	29.83114
10D	52.33333	28.16667	46.17834	109.48	65.68	40.00731

Figure 6a shows the performance for single key query of C2A and TMA based algorithms for varying number of dimension and Fig. 6b shows the average improvement of C2A based algorithm over TMA based algorithm for single key query. The improvement is nearly linear with incresing number of dimension. Figure 7 shows the retreival performance when the known or query dimension is on row wise (Fig. 7a) and column wise (Fig. 7b) for C2A based algorithm. The query was set such that only a row (or column) for C2A is selected. The comparison witth row wise and column wise query is shown in Fig. 7c. The row wise retrieval has improved performance than of column wise retrieval. This because the compiler maintains the row wise data layout in the system. Therefore, when the retrieval is performed by fixing rows for varying columns, it increases the cache hit rate of the processor because of the data locality. Figure 8a shows the range key query performance for varying number of dimension and Fig. 8b the improvement of C2A based algorithm over TMA based algorithms. The C2A based algorithms has improved performance than TMA based algorithms. Therefore, we conclude that the C2A based algorithms has improved performance than the TMA based algorithms for retrieval operations on higher dimensional arrays. Table  1 shows time comparison of single key and range key query for C2A based algorithms.

## Related works

Multidimensional data have been well studied in the form of the multidimensional arrays such as ArrayStore^16^, SciDB^2^, TileDB^6^, ChronosDB^17^ etc. Many parallel processing workloads for arrays are supported by ArrayStore^16^. An array storage manager is provided by TileDB^6^ manages the dense and sparse with embeddable libraries. A distributed array database is provided by ChronosDB^17^. All the array models use the TMA as their basic data structure and hence the retrieval is based on the TMA algorithms. To improve array computation^18^, introduced the EKMR scheme, which consists of a set of two-dimensional arrays that represent a high-dimensional array. They used the K-map technique to transform a four-dimensional array to a two-dimensional array. A hierarchical structure including an array of pointers is the *n* ($n>4$) dimensional generalization of EKMR. For large values of *n* ($n>4$) there are $n-4$ pointers arrays required^19^. Proposes GPU-based automatic data layout alterations for structured grid codes with dynamically generated arrays^9^. Proposes a loop transformation-based strategy for improving data locality in multidimensional arrays. They showed how transformation can help with array operations. In order to facilitate access to the elements, chunking, reordering, redundancy, and segmentation of large arrays are proposed in^12^.To improve speed^20^ suggest chunk-by-chunk caching. Chunking of arrays is the technique of breaking huge multidimensional arrays into smaller chunks for storage and processing. Each chunk is a *n*-dimensional array with a shorter length than the original array. Ref.^16^ demonstrates a chunking strategy for storing and analyzing multidimensional arrays, with the chunks remaining n-dimensional. The multidimensional query is well defined for datacube computation as MOLAP operations^11^. A good data structure is required for efficient datacube construction, which has been identified as one of the most critical and essential issues for MOLAP^21,22^. A data structure for growing data is proposed in^21^ and show the superiority of the structure over TMA data. The virtual denormalization for the main memory OLAP is presented in^22^ and shows some superiority of the scheme using TMA. Multidimensional data points are mapped to one-dimensional data points in^23^ for query operations. A new query problem namely k-truss most favorites querying problem is defined in^24^ to retrieve the most favourite object with users’ preferences based on the top-t favorites query. To reduce the query computation space and improve the query efficiency they also develop an optimized reverse query algorithm. To speed up query processing time and improve query accuracy of Bloom filter (*BF*) a novel sequence-based Bloom filter($B_{h}BF$) is proposed in^25^ which also support four important operations like insertion, query, deletion, and update. A probabilistic reverse top-k queries for monochromatic and bichromatic cases over uncertain databases are proposed in^26^ with effective pruning heuristics to reduce the search space. A comprehensive survey on personalized graph queries to compute personalized query results for users on the basis of their personalized preferences is presented in^27^. A scheme is developed to answer multidimensional range queries on multidimensional data using bucketization in^28^. The bucketization is treated as an optimization problem to reduce the risk of disclosure keeping the computational overhead below a certain overhead. In this paper, we convert the *n* dimensional data points to 2-dimensional points. The array models described in this section use TMA as their basic data structure, but the proposed C2A based algorithm shows better retrieval performance than the TMA.

## Conclusion

We propose and evaluate an effective algorithm for query processing. Our algorithm is based on a converted multidimensional array. We calculate the execution time for single key and range key queries for TMA and C2A. The performance of our proposed C2A based algorithm outperforms the TMA-based algorithms. The reason for the better performance is that C2A requires two loops only that increases cache hits. The approach may easily be applied in a parallel and distributed environment for parallel processing, which is an essential future path of the work. The MapReduce algorithm can be developed for the efficient processing of multidimensional array data. The scheme can also be connected to compress database applications for scanty information. We believe the proposed retrieval algorithm using converted array can be efficiently applied to higher dimensional array data processing for actual applications.

## Data Availability

The datasets that are used in this experiment are of the following two sources. The 4D data set is a 4-order tensor with numeric data values. The experiment on 4D data is done with different data sets from the Formidable Repository of Open Sparse Tensors and Tools (FROSTT)^29^. FROSTT is a collection of publicly available sparse tensor datasets and tools. It can be found at http://frostt.io/tensors. The 6D, 8D and 10D data sets that we used in this experiment are generated automatically using the *rand*() function of gcc compiler. The datasets can be available from the corresponding author on reasonable request.
